# Co‐Creating and Refining a Values Assessment Tool (VAsT) for Women With Metastatic Breast Cancer

**DOI:** 10.1002/pon.70173

**Published:** 2025-05-08

**Authors:** Lorinda A. Coombs, Victoria Crowder, Madison Black, Kelly Tan, Emily M. Ray, Renée M. Ferrari, Erin E. Kent, Daniel S. Reuland, Ashley Leak Bryant

**Affiliations:** ^1^ University of North Carolina at Chapel Hill School of Nursing Chapel Hill North Carolina USA; ^2^ Lineberger Comprehensive Cancer Center Chapel Hill North Carolina USA; ^3^ University of Pittsburgh School of Nursing Pittsburgh Pennsylvania USA; ^4^ University of Pittsburgh Medical Center Hillman Cancer Center Pittsburgh Pennsylvania USA; ^5^ Division of Oncology Lineberger Comprehensive Cancer Center University of North Carolina at Chapel Hill School of Medicine Chapel Hill North Carolina USA; ^6^ University of North Carolina at Chapel Hill Gillings School of Global Public Health Chapel Hill North Carolina USA; ^7^ Division of General Medicine and Clinical Epidemiology & Lineberger Comprehensive Cancer Cente University of North Carolina at Chapel Hill School of Medicine Chapel Hill North Carolina USA

**Keywords:** cancer, community advisory board, metastatic breast cancer, oncology, qualitative, quality of life, shared decision‐making, values assessment

## Abstract

**Background and Aims:**

Patients with metastatic breast cancer (mBC) and their families often have differing perspectives on treatment goals. This highlights the need for systematically eliciting patients' and care partners' values to ensure values‐aligned treatment decisions. This study aimed to inform the development of a values assessment tool to facilitate communication of priorities and preferences with oncology clinicians.

**Methods:**

Two rounds of semi‐structured interviews were conducted with women with mBC from the Southeastern and Northeastern U.S. Recruitment included at least 50% of participants identifying as African American/Black, Latinx, Asian, American Indian, or Native American.

**Results:**

The initial round of 13 interviews yielded eight candidate domains. After confirmatory interviews with additional participants, the researchers identified nine final domains relevant to treatment decisions for mBC: desire not to appear sick; desire to help other women with breast cancer by participating in clinical research; financial concerns; living to care for a loved one; maintaining sexuality; maintaining quality of life; maximizing time away from medical appointments; minimizing and managing side effects; and slowing disease progression with an effective treatment.

**Conclusion:**

Eliciting treatment decision values across multiple domains and effectively communicating them with clinicians is a crucial aspect of patient‐centered care to align values with care goals. To help patients identify and express their values to clinicians, we are developing a values assessment tool specifically for mBC. Future research will pilot this tool to assess its impact on communication between clinicians and patients and health outcomes for women with mBC.

## 1 Background

Metastatic breast cancer (mBC) affects more than 40,000 women in the United States (U.S.) each year [1]. Because of improvements in treatment, patients with metastatic breast cancer are living longer, with 34% living for five years or more [2]. Although metastatic breast cancer remains incurable, treatments can prolong survival beyond the previous two‐to‐three‐year average [3, 4]. Because of the increasing number of available therapies, decisions around selecting treatment rely heavily on available data regarding efficacy and balancing treatment toxicities, comorbid illness, and patient preferences.

Patients with mBC have reported different treatment priorities than those with curable breast cancer; consequently, considering their unique perspectives is critical to ensuring treatment is consistent with their values (what is most important to them within the context of their diagnosis) and goals [5]. Oncology clinicians often make direct treatment decisions without systematically eliciting patient input, assuming longevity is the patient's most important goal [6]. Studies have shown that clinicians infrequently elicit patient preferences in medical decision‐making [7, 8]. This omission will become increasingly critical as women are living longer with mBC and will have multiple treatment options to decide upon. Patients and their families often have different perspectives from their oncology clinicians [9], underscoring the importance of systematically eliciting the values of patients and their families to ensure value‐aligned treatment decisions.

A metastatic cancer diagnosis is a significant event for the person diagnosed as well as their family and caregivers, with some studies suggesting that a cancer diagnosis may have a similar or more significant impact on caregivers [7, 10]. Caregivers of patients with mBC are a fundamental source of patient support. They are increasingly involved in treatment decisions [8], highlighting the need to elicit their input when selecting a cancer treatment.

A standardized approach to values assessment in mBC does not currently exist. Existing values assessment tools have been developed for serious illnesses in tertiary care settings [11] and include cancer, among other comorbidities, in the ambulatory environment [12]. However, when these tools, the Graphic Values Tool [11] and the Short Graphic Values Tool [12] were previewed with patient advocacy groups as part of this research, many survivors felt that the tools had limited applicability to their metastatic breast cancer because of differences in the patient groups, for example, women's concerns regarding sexuality, identity, and caretaking were not represented, or the options did not speak to their values. As a result of this input, the researchers determined that co‐creating and developing an mBC‐specific values assessment tool was essential to meet the needs of women with mBC specifically.

Incorporating values into shared decision‐making is especially important for women who identify as African American/Black, Latinx, Asian, American Indian, or Native American. Studies suggest that these groups are at risk for poor communication in prognosis, higher rates of metastatic disease, and worse cancer outcomes when compared to non‐Hispanic White adults [13]. Black/African American women living in the South have the highest breast cancer mortality rate [14, 15] and also experience treatment delays compared with other women with breast cancer in the U.S. [16].

Tools that support values communication are valuable for patients, caregivers, and clinicians because they support conversations that address patients' and caregivers' uncertainty around treatment risks and benefits and facilitate treatment decisions. Because patients from minoritized vulnerable groups are more likely to be diagnosed with metastatic cancer and are at significant risk for poor communication in prognosis and treatment options, they are poised to benefit from an intervention that elicits patients' values and supports communication with their oncology clinician. To address this missing critical component of mBC care, the authors conducted a qualitative study to inform the development of a values assessment tool (VAsT) to support the communication of patient values for women with mBC.

## 2 Methods

A qualitative study was conducted to identify domains for the values assessment tool. Recruitment goals included ensuring that at least 50% of participants identified as African American/Black, Latinx, Asian, American Indian, or Native American. The University of North Carolina at Chapel Hill (UNC‐CH) Institutional Review Board (IRB) and the UNC‐CH Lineberger Comprehensive Cancer Center Protocol Review Committee reviewed and approved the study (IRB 22–1806).

### 2.1 Positionality Statement

The University of North Carolina Lineberger Comprehensive Cancer Center Connected Health for Applications and Interventions (CHAI) Core qualitative team conducted the first round of interviews with eligible participants. The CHAI Core interviewers came from academic research backgrounds and had extensive experience conducting qualitative interviews. The principal investigator (LC) conducted a second round of four confirmatory interviews. Dr. Coombs's background is both clinically and research focused. She has extensive clinical experience as a nurse practitioner and has conducted qualitative interviews with patients in clinical settings.

Interviewers did not have prior relationships with participants before they were enrolled. Participants were informed of the study's objectives, interviewers' backgrounds and roles, and the principal investigator's research interests. Interviewers additionally sought to improve participants' comfort by starting with an open‐ended question to elicit conversation.

### 2.2 Participants

Adult women diagnosed with mBC were recruited through outreach to community organizations and ambulatory oncology clinics from two comprehensive cancer centers, one in the Southeastern U.S. and the other in a Northeastern urban area. Inclusion criteria were: (1) Diagnosis of mBC; (2) age≥18 years; (3) ability to understand and read English; and (4) ability to understand and participate in study procedures. Individuals were excluded if they could not provide consent, communicate verbally, or were enrolled in hospice care. Twelve to fifteen interviews were planned to identify and confirm the value domains.

### 2.3 Tool Co‐Creation With Community Guidance

A Community Advisory Board (CAB) was established to guide VAsT development. Recruitment for the CAB members included two patient participants: women with mBC, both African American/Black, one care partner to a patient with mBC who is White, and a breast oncology clinician (medical oncology physician who is also White). The CAB met approximately once per month for an hour via video platform, and the caregiver and patient participants were compensated for their time; the oncology clinician was not. During meetings, CAB members were updated on the VAsT progress, reviewed the most recent tool, and advised on tool content and usability. Feedback from the CAB resulted in changes to the tool, including adding a domain, reviewing missing items, and approaches to ensure racial and ethnic diversity was represented in the tool. The CAB also provided guidance on communicating the tool's results with clinicians. Conversations were instrumental in developing the VAsT as members of the CAB provided real‐world experience throughout the development of the tool and critical perspectives that contributed to the research.

### 2.4 Data Collection and Analysis

Three experienced qualitative researchers (two women and one man) affiliated with the UNC CHAI Core conducted the initial interviews. The initial participant interviews followed a semi‐structured interview guide (Appendix A) developed through an iterative process with the principal investigator, the VAsT study team, and the CHAI Core qualitative research team (comprised of two women and one man completed the initial interviews) to elicit insights and descriptive details from the participants' perspective. Upon completing the interview, participants received a $75 gift card.

Recruitment continued until thematic saturation was reached. After reviewing the initial interviews, an independent qualitative expert (RMF) conducted a second evaluation of the findings. The principal investigator (LC) performed confirmatory interviews with previously interviewed participants selected for demographic representation, and each expressed a desire to remain involved in the project. Four additional one‐on‐one interviews were conducted, along with further input from two separate meetings with two patient advocates participating in the community advisory board (CAB). All interviews were held via phone or a meeting platform, audio‐recorded, professionally transcribed verbatim, verified, and uploaded to Dedoose software for data analysis [17]. Transcripts were reviewed for consistency and de‐identified before being entered into Dedoose.

Research team members collaborated to develop an initial codebook based on interview questions and notes taken during interviews. The research team and CHAI Core qualitative interviewers tested the initial codebook and independently coded several transcripts to allow additional fine‐tuning of concept definitions and revising decision rules. This process continued until coding was replicated across two coders. When an emergent theme or discrepancy was identified, it was reconciled through extensive discussion with a third or fourth coder. A matrix was created in Microsoft Word to organize relevant participant quotations, notes on the identified domains (e.g., whether quotations support the retention, deletion, or revision of a domain), and critical attributes.

Thematic analysis identified domains and themes, and codes were applied to describe the (a) context and history of participants' breast cancer, including perceptions of the cancer care team; (b) factors that influence the treatment plan and decision‐making, such as what was most important when deciding the treatment plan; and (c) participants' experience communicating around treatment plan decision making, such as how participants communicated their values with clinicians, and if this impacted the treatment plan in any way [18].

After identifying the initial domains, confirmatory interviews were conducted with four participants to ensure all relevant domains were included. During these interviews, participants were informed about the domains and asked to reflect on each one, identify which domains were essential to them in the context of their mBC diagnosis and treatment, and determine if any areas were missing from the list (Appendix B). This process followed established protocols for cognitive interviews and tool development [19, 20].

## 3 Results

Between January and June 2023, fourteen women with metastatic breast cancer (mBC) were approached to obtain 13 initial interviews, as one individual declined participation. Follow‐up confirmatory interviews were conducted with four participants in February 2024 (see Table 1). All participants were female, with an average age of 55.5 years (ranging from 40 to 76 years). Six participants (46%) identified as Black or African American, Native American, or multi‐racial. Most participants had received a mBC diagnosis within 1–3 years (*n* = 5, 38%) or 4–6 years (*n* = 4, 31%) at the time of the interview. Initial interviews lasted between 45 and 60 min, while follow‐up interviews lasted 25–37 min.

**TABLE 1:** Participants demographic.

Characteristic	*N* (%)
Age (mean, range)	55.5 (40−76)
Age (years)	
40−49	5 (38%)
50−59	4 (31%)
60−69	2 (15%)
> 70	2 (15%)
Diagnosis length, years (mean, range)	4 (0.5−11)
< 1	1 (7.5%)
1−3	5 (38%)
4−6	4 (31%)
> 6	2 (15%)
Unknown	1 (7.5%)
Origin of participants for original interviews	
Southeastern US	9 (69%)
Northeastern US	4 (31%)
Origin of participants for follow‐up interview	
Southeastern US	2 (50%)
Northeastern US	2 (50%)
Sex	
Female	13 (100%)
Race	
White	7 (54%)
Black or African American	4 (31%)
Native American	1 (7.5%)
Multi race	1 (7.5%)
Ethnicity	
Not Hispanic or Latino	11 (85%)
Hispanic or Latino	2 (15%)
Sexual orientation	
Straight/Heterosexual	12 (92%)
Bisexual	1 (7.5%)
Marital status	
Married/Partnered	7 (54%)
Single	2 (23%)
Divorced	2 (15%)
Separated	1 (7.5%)
Employment status	
Full‐time	5 (38%)
Part‐time	1 (7.5%)
Unemployed	2 (15%)
Retired	3 (23%)
Other	2 (15%)
Highest level of education	
Some college, no degree	4 (31%)
College and/or advanced degree	9 (69%)
Annual household income	
$35,000−$49,000	4 (31%)
$50,000−$74,999	2 (15%)
> $75,000	5 (38%)
Prefer not to answer	1 (7.5%)
Don’t know	1 (7.5%)

Seven domains were initially identified: (1) social support, (2) communication, (3) treatment options, (4) finances, (5) cultural influences, (6) values, and (7) religion/spirituality. Confirmatory interviews identified a missing domain, “sexuality,” and clarified specifics regarding the other domains. For example, the importance of participating in clinical research was explicitly identified as a way to offer support to other women with mBC. After a presentation to the community advisory board (CAB), an additional domain, “maximizing time away from medical appointments,” was also identified, resulting in nine final domains.

The final significant domains were: (1) the desire not to appear sick; (2) the desire to assist others by participating in clinical research; (3) concerns about the affordability of cancer care; (4) the motivation to live for a loved one; (5) the need to maintain sexuality while minimizing the impact on intimate relationships; (6) the importance of maintaining quality of life during treatment; (7) maximizing time spent away from medical appointments; (8) managing and minimizing the side effects of treatment; and (9) stopping or slowing disease progression through an effective treatment plan that includes the patient.

### 3.1 Revised Values Assessment Tool Domains

**FIGURE 1:**
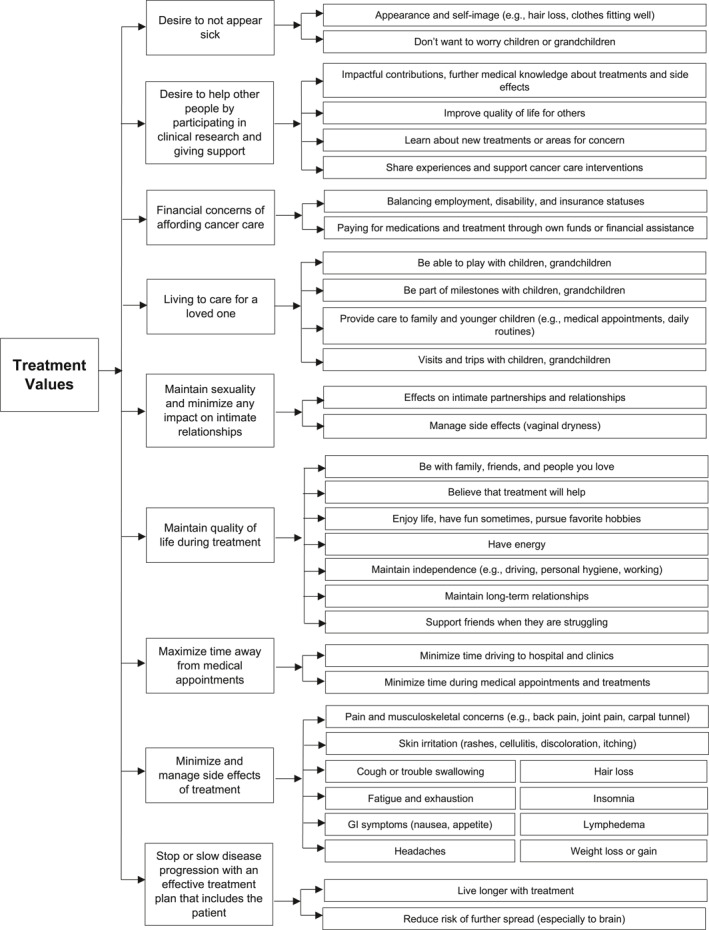
Domains and key attributes regarding values in treatment decision‐making for women with mBC. Adapted from McCormack et al. (2011).

Nine final domains regarding patient values for oncology treatment decisions and critical attributes were identified. (Figure 1) The themes for each domain are presented (Table 2).

**TABLE 2:** Domains, themes, and exemplar quotations regarding values in oncology treatment decision‐making from women with metastatic breast cancer (*N* = 13).

Domain	Theme	Exemplar quotations
1. Desire to not appear sick	– Not worrying their families – Patients want to “live life’	*“Even if I don't feel well, I won't give off the appearance of a sick person. That's held true. I don't think I look sick. I'm sure that will probably change, but at least for now, the important things to me was I didn't want to be a burden. I didn't wanna look sick, and I wanted to be there for my family in all of the ways. That was what was important to me.” [ID01]* *“…that's a big thing to me. Because with my medications, my hair did fall out. Not completely. But it thinned and I had many bald spots. Now is starting to grow back, and I don't know why. But yes, that is a big thing to me because cancer does not define me. I am not cancer. I just have cancer.” [ID07]*
2. Desire to help other people by participating in clinical research and giving support	– Making impactful contributions in the lives of others – Opportunity to learn more about cancer care	*“I've noticed a difference from 20 years ago and now. And a lot of that is because the people was willing to participate in clinical trials. Because that's the only way that you'll know. If people participate, that's the only way that you'll know if things work or not.” [ID04]* *“I just hope that this helps someone else. I really, really, do. I don't have to put on airs or put on shows because this is my life, and this is my story. This is real, and this is who I am, and this is how I feel about what I'm going through. I always say everybody's going through something, and I'm not minimizing anybody else's. Like I say, my pain is my pain, be it internal or external or internal and it comes out externally, but I can go through and grow through this process. It'll either make me better or it'll make me bitter, and I'm not letting it make me bitter, so it has to make me better, if you hear what I'm saying.” [ID12; Discussing participation in a cancer care intervention]*
3. Financial concerns of affording cancer care	– Uncertainty with information about financial assistance throughout treatments – Changing employment, disability, and insurance statuses	*“I'm goin’ through the finances now because I haven't worked since August. I racked up some financial bills and they're callin’ me, and they denied me financial assistance because they go by your income for last year. Well, last year I was perfectly healthy.” [ID04]* *“…‘Yeah, I'm sitting here because insurance pays you for me to be here, but if I don't work, I might not get the same treatment package.’ He will agree with me, so we'll discuss, ‘Okay, what can you do? Can you work from home? Can you work part‐time? How does that affect your finances and all that?’ We go through the process of what I can and cannot do and how will that impact my quality of life.” [ID10]* *“I learned from a social worker‐type person that there was programs that we might have qualified because we were of certain income or whatever. Then the pharmaceutical companies, I got help from them. That was offered. Had this particular person not told me about it, I would have continued to have paid it because I didn't know any better. That wasn't offered from anybody else. We didn't receive a paper.” [ID13]*
4. Living to care for a loved one	– Responsibility of caring for children – Enjoying time with children, grandchildren, and other loved ones	*“I ask God, I'm like, “I really need to be here a little while. I want to be here long enough to raise my son.”” [ID03]* *“My main concern, and my main prayer, was because I have lymphedema in my right arm; so, I'm not able to lift things a lot in my right arm. And I had a lot of weakness in my right arm. So, my main prayer was: “Will I be able to lift him? Would I be able to play with him? Would I be able to comb his hair?” because I'm right‐handed.” [ID04]* *“That was my I have to beat this ‘cause I've got grandkids ‘cause my kids are old enough to look after themselves. My grandkids will get looked after by their parents, but as a grandmother, we all wanna be there, most of us wanna be there for our grand kiddies.” [ID06]* *“…for me right now, it's just for my youngest son; because I kind of feel like nobody gets him the way I do, so I can't just not be there for him.” [ID11]*
5. Maintain sexuality and minimize any impact on intimate relationships	– Managing side effects related to sexual health – Affecting intimate relationships	*“But then it's affecting marriages; and then if your marriage is not going good, it's affecting your overall quality of life.” [ID11]* *“I mean, I wouldn't want to carry on a long conversation about it because it is private. But I feel like it would have been beneficial going into all this, to have had a little talk about how it would change.” [ID07]*
6. Maintain quality of life during treatment	– Enjoying experiences, memories, and daily life	*“I think my biggest, out all of my questions I do remember, were really just like “Day‐to‐day how is this going to affect me?” Not so much “Am I gonna die in a year? Am I gonna die in two years?” It was “What do I expect in the immediate future after starting this?”” [ID01]* *“I can eat anything I want to. I go to church every Sunday. You know what I'm saying? I go out with my girlfriends and do whatever—go have a glass of wine, go out to dinner, things like that—that if I was on IV chemo, most likely I wouldn't be able to. Once you start to feel better from your treatment, it's already time for you to start your next treatment.” [ID03]* *“Well, I think quality of life is being able to enjoy most days and being able to be with the people that you love and that love you and being able to have fun sometimes.” [ID07]*
7. Maximize time away from medical appointments	– Minimizing effects on financial concerns Improving quality of life	*“Well, the current decision, I don't wanna go every month, so going to the doctor every month and getting shots every month, that was my main concern, how it made my body feel every month getting stuck. I didn't wanna go every month. I believe once I had my second opinion that there were different options that were spoken about as well.” [ID08]*
8. Minimize and manage side effects of treatment	– Personal experiences with side effects – Balancing with effective treatment and quality of life	*“All those pills. I take ‘em for a bit, and then I'd have a reaction, and I'd have to go to another one, then I'd have another side effect, and I'd have to go to another one. I had carpel tunnel; I had trigger thumb. I was just gettin’ all these weird things.” [ID06]* *“Well, it started out every—I was going every week. I think at one point when I was doing radiation, I was going every day. To go from that to every month, it was okay with. The problem started when I started to feel pain in where I kept getting the shots repetitively. It was fine at first, but then your body can only take so much.” [ID08]* *“It always does [affect treatment decisions], because every drug has a whole long list of side effects that affect each person differently.” [ID11]*
9. Stop of slow disease progression with an effective treatment plan that includes the patient	– Trying new treatments – Emotional toll	*“That's the statement that I think about often is how they wanna treat it like a chronic disease because that gives a person hope. Like, “Okay, this is gonna be something we're just gonna just keep treating it. They have all sorts of things they can throw at it.” By saying that, they're saying, “Treat it like a chronic disease,” it's pretty wild how that's helped me get through some tough times.” [ID01]* *“As far as actually making decisions on my treatment, it's like okay, the cancer's acting up, where do we go now.” [ID02]* *“In my mind, the more chemo that I take, the 600 Kisqali, I didn't want to stop taking 600 Kisqali. My oncologist had recommended it, from when she's seen my fatigue. But my thing was the more oral chemo I take; the less chance I have of it spreading to my brain or my bones.” [ID04]*

#### 3.1.1 Desire to Not Appear Sick

A significant value identified was the “*Desire not to appear sick*.” Participants described this as how they physically looked and determined that it impacted their relationships with loved ones, and not appearing sick enhanced their quality of life. When discussing the desire not to appear ill, a primary concern was *not worrying their families*, especially younger children. They felt that looking “*normal”* and maintaining an appearance of feeling well and having energy made their younger children's experiences of their mBC diagnosis easier. Their specific concerns included treatment‐related hair loss. Additionally, while some participants described support from family with appearances (e.g., family members shaving their hair in solidarity), some noted tension related to not appearing sick and, therefore, having others not understand the severity of the side effects or impact of the treatments.

Participants expressed that not looking sick encouraged them to engage in activities they might not have otherwise undertaken. The connection between participants' appearance and what they felt they could do was seen as motivation to participate in more activities. Participants also recognized the link between looking healthy and being immunocompromised or experiencing side effects, which was more often discussed in the context of chemotherapy.

I'm trying just to live life and enjoy every single day. Maybe it's because I'm not sick. You know, I’m not sick. I don't look sick. I look very healthy, all that.

[ID03]

#### 3.1.2 Desire to Support Other Women by Participating in Clinical Research

A second essential value identified was the “*Desire to help others by participating in clinical research and providing* *support*.” This included clinical drug trials and cancer care interventions (e.g., symptom monitoring and supportive measures). Overall, participants aimed to make *significant* contributions to enhancing the health and quality of life of individuals with cancer. Specifically, they desired to improve understanding of new treatments, enhance supportive care interventions, and elevate the quality of different patients' experiences with medical treatments.

Additionally, by participating in research, participants identified the opportunity to learn more about cancer care. Participants were interested in learning about new treatments, supportive care, and the most up‐to‐date information about cancer care. One participant emphasized the importance of awareness about research in supportive care:

So, it's just not clinical trials; there's also clinical research that can be done to help women like me. Other women need to know that that's there for them, and that's important as well.

[ID11]

#### 3.1.3 Financial Concerns of Affording Cancer Care

A third significant value identified was “*Financial concerns about affording cancer care*.” Participants described how they selected or changed treatment plans based on their financial circumstances and navigated finding information and financial assistance. Many participants described uncertainty with information about financial aid throughout the treatments. As treatment options changed, one participant noted that a social worker was the only person to provide continued and updated information on available financial assistance. Participants also expressed concerns about finding further financial aid grants after using up their one‐time options. While some participants expressed relief and satisfaction with their financial situation, many identified ongoing challenges related to finding ways to pay for medical appointments and treatments.

Participants frequently discussed challenges with changing employment, disability, and insurance statuses. Complicating circumstances included switching insurance from a state insurance plan to federal (e.g., Medicare), changing from full‐time employment to part‐time, or applying for disability. This affected treatment decisions as participants discovered that treatments may not be covered under a new insurance plan, or switching to a new treatment plan may be more expensive and not covered under their deductible. Participants also experienced challenges with changes in their income, such as going on disability:

I've looked at going on social security disability, but of course…I'd have to stop work, and I would have to wait six months, the requisite waiting period, before I ever got my first check. So, how am I supposed to live in those six months? I don't have six months' worth of savings. Yeah, you look at different options, and you always come back to, “do I have enough money to do this?’

[ID10]

#### 3.1.4 Living to Care for a Loved One

A fourth significant value identified was “*Living to care for a loved one*.” This was primarily discussed within the context of caring for children, grandchildren, or parents, among other loved ones. They addressed the responsibility of caring for others, the joy and fun of caring for grandchildren, and the concern over who else would take responsibility when they were the primary caregivers. For some, the connection and care with their children were unique, and parents worried that other caregivers might not be quite the same or “get” their children the way they do. Participants discussed their treatment decisions as facilitating the activities they enjoy with loved ones. Some brought humor and laughter to the discussion, for example:

I’m gonna be honest. That was it. That was like, “I wanna live to see my grandchildren terrorize my son.”

[ID06]

#### 3.1.5 Maintain Sexuality and Minimize any Impact on Intimate Relationships

A fifth significant value identified was “*Maintaining sexuality and minimizing any impact on intimate relationships*.” Many women with mBC expressed the importance of sexuality to their health, relationships, and quality of life. Specifically, participants frequently wanted side effects related to sexual health to be managed by their treating oncology clinician, such as caring for symptoms like vaginal dryness. Participants mainly received information on symptom management from third‐party organizations targeted toward cancer care and expressed frustration with this and a desire for more information from clinicians. Conversations with clinicians were aimed at managing complications, as one woman described a conversation with her clinician:

I don’t think that it was a good idea for you to wait for me to come in with the symptom of a UTI because of vaginal dryness. I think that it would’ve been important for you to explain this to me to show me what—to tell me what to do to prevent that from happening.

[ID11; Speaking about a conversation with clinician]

When describing sexuality, participants also described how mBC was affecting their intimate relationships. Considerations for intimate relationships included changes to sex and intimacy and the possibility of adverse effects on their relationships with spouses and partners, which affected their quality of life.

#### 3.1.6 Maintain Quality of Life During Treatment

The sixth significant value identified was to “*Maintain quality of life during treatment*.” With this, all participants identified the goal of *enjoying experiences, memories, and daily life.* Many discussed the daily routines with their loved ones and children and wanted to make positive and joyful memories with them. Participants defined “enjoying” their lives as not having side effects such as pain or fatigue and continuing to accomplish their favorite experiences. For example, one participant describes her favorite mountain biking spot:

After I was diagnosed, I was really like, “Oh my gosh, I'm never gonna be able to get on my bike again. I'm certainly not gonna make it to my favorite”—there's a spot on the mountain where I mountain bike a lot that has always been a challenge to get to, but I've always been able to do it. I remember thinking that I'm not gonna make it to that spot. For the first few months after starting treatment, I was not able to. I would get on my bike, and I would only make it a third of the way. Then it was half the way. Finally, I did make it back up, and I continue to do that.

[ID01]

Women with mBC consistently identified quality of life as the main driver for treatment decisions, saying, for example, *“If you don't have quality of life, you don't have anything.”* [ID04]. Many participants changed their treatments based on their enjoyment of experiences and level of functioning, with symptoms and side effects. While some described the necessity of maintaining daily routines and work, participants also expressed the importance of enjoying life and experiences because of their diagnosis.

#### 3.1.7 Maximize Time Away From Medical Appointments

The seventh significant value identified was to “*Maximize time away from medical appointments*.” This value was expressed as a separate concern, in addition to minimizing financial concerns and improving quality of life. Many participants traveled significant distances to their medical appointments and for treatment. This value was identified with input from caregivers and included the costs of driving or taking taxis, time spent away from work or family, and other concerns, such as finding childcare. In this way, maximizing time away from medical appointments minimizes the effects on financial matters. Additionally, participants described that less frequent treatments and clinic appointments could improve their time spent with loved ones and thus improve their quality of life.

#### 3.1.8 Minimize and Manage Side Effects of Treatment

The eighth significant value identified was to “*Minimize and manage side effects of treatment*.” Learning the expected side effects of different treatment options was recognized as essential and typically was present when good clinician communication occurred. Participants identified discussion of side effects as crucial in making treatment decisions; however, many also described the importance of monitoring their symptoms over time because their personal experience of side effects varied. Not everyone experiences the same side effects from each treatment, and many women described either experiencing an unexpected side effect or developing new symptoms over time.

Several participants described continued changes to treatment plans to manage side effects and improve their functioning in daily routines and enjoyment of life experiences. This was informed by the clinician's expertise in treatment effectiveness and toxicity management, and the participants considered side effects, quality of life, and efficacy together. As one participant described:

I think we both have that understanding that I want to do whatever I can to hang in there and have a good quality of life. As long as I’m the one that’s—I think the patient needs to be the one that determines what quality of life. If I want to go through those side effects, that’s my choice.

[ID02]

#### 3.1.9 Stop or Slow Disease Progression With an Effective Treatment Plan That Includes the Patient

The ninth value identified as important was *“Stop or slow disease progression with an effective treatment plan that includes the patient*.” Participants emphasized that patient preferences and values needed to be included in discussions about treatment effectiveness. Many expressed a desire to try *new treatments* because they wanted to live, but they balanced this with concerns about side effects and quality of life.

Along with the effectiveness of treatments, women discussed the heavy *emotional toll* this could elicit. The difficulty of making treatment decisions was discussed, including hope for effective treatments, fear of ineffective therapies, no cure, and the fear of the unknown when initiating treatment. One participant highlighted the emotions surrounding changes to the treatment plan:

Emotional to think that—for whatever reason in my personal brain, having to change treatment seems like a weird big milestone—I don't know, somehow dictates some miraculous—some end calculation of how much time there is left.

[ID09]

#### 3.1.10 Suggestions for VAsT Implementation in Practice

Key components identified during the interviews included guidance on when the information gleaned from the tool would be most valuable (at any time during diagnosis and treatment); participants recommended completing the tool before meeting with their clinician and having a record of their past rankings to help clinicians and care partners understand the evolution of their values over time (and with different treatments). For example, the tool can reassess values when making treatment decisions after a routine scan or when progression is identified. Another critical suggestion from the participants and CAB was the need for an area on the tool for additional thoughts or a specific value not identified on the tool.

## 4 Discussion

This study identified several key findings from a diverse participant group of women, including developing a comprehensive list of values important in treatment decision‐making. Nine domains were identified as significant to participants with metastatic breast cancer during treatment decisions: (1) desire to not appear sick; (2) desire to help others by participating in clinical research and giving support; (3) financial concerns about affording cancer care; (4) living to care for a loved one; (5) maintain sexuality and minimize any impact on intimate relationships; (6) maintain quality of life during treatment; (7) maximize time away from medical appointments; (8) minimize and manage side effects of treatment; and (9) stop or slow disease progression with an effective treatment plan that includes the patient.

Values were elicited as a first step in creating a values assessment tool (VAsT) to facilitate communication during treatment decision conversations between patients, care partners, and clinicians. Participants were enrolled in the study at varying times during their survivorship and treatment. This enhanced our study's applicability to the diverse care trajectory, given the prolonged survival of women with mBC and the potential for shifting values over time [4]. In the interviews, participants supported this by expressing how their values have shifted over time, including an emphasis on quality of life. The values assessment tool developed from this study has the potential to enhance the communication of values with patients, care partners, and clinicians, and we will next pursue further analysis of the tool's impact on communication, patient satisfaction, and value‐concordant care.

Of note is that the study's qualitative interview methods revealed the importance of the researcher's gender in interviews with women with mBC. In the initial round of interviews with a male qualitative researcher, not one participant mentioned sexuality as an essential value in their treatment decisions. However, when confirmatory interviews occurred with the PI (a female researcher), multiple respondents identified sexuality as critically important in their values and treatment decisions. The CAB members also echoed the importance of sexuality, especially if someone was in a newer relationship when they were diagnosed. The influence of the interviewer's gender is not well‐described in qualitative research on women with mBC [21]. Future qualitative studies may want to consider the interviewer's gender when interpreting findings.

### 4.1 Clinical and Research Implications

The development of the data‐driven tool (VAsT) is essential to improving patient communication and patient‐centered treatment decision‐making in clinical practice. Specifically, it is necessary to evaluate its use for women with mBC who identify as Black/African American, Latinx/Hispanic, or American Indian/Native American to enhance communication, for whom prior research suggests may be the recipients of poor communication during prognosis discussions [13]. Historically marginalized women with mBC are poised to benefit from a tool that promotes value‐based communication to clinicians. Future studies will evaluate the value of communication tools on patient‐provider communication and the impact on treatment decisions and health outcomes, such as anxiety and depression [22, 23].

### 4.2 Strengths and Limitations

The final tool will be the product of multiple critical evaluations and knowledge checks with participants and the CAB. The study's original goal was to ensure that the findings represented a diverse group of women with mBC. Additionally, the active participation of the CAB members in the co‐creation process enhanced the rigor of the research. It ensured that the final version of the tool would reflect multiple perspectives. Values‐based decision‐making is complex; this study is an initial attempt to identify the domains. However, further user experience research is needed to make this tool usable for patients and clinicians in clinical settings. Our next steps include ensuring that the tool supports a positive user experience and assessing any user assumptions that may conflict and reduce the benefit of the tool.

Previous literature on values‐based decision‐making focused explicitly on a treatment decision. This study's inclusion of all values makes it a more holistic examination of what is important to women with mBC, other than life prolongation.

Limitations of the study include the relatively small number of participants and a lack of representation of sexual and gender minority voices. Only one participant identified as bisexual, and all others identified as heterosexual. Additionally, all participants had completed high school and received some college education.

Similarly, the current version of the tool has limited generalizability to other populations and cancer types. In our initial investigation of the utility of previously existing values assessment tools, such as the Short Graphic Values Tool, patient advocacy groups identified that it lacked relevance to them. This suggests that there are specific values considered for different populations, cancer types, and countries (the SGVH was developed in Canada).

## 5 Conclusion

Women with mBC must identify values when making treatment decisions and communicate these with their care partners and clinicians to ensure that care is aligned with their values and preferences. We used qualitative interviews with a diverse group of women with mBC to create a comprehensive list of values to inform the development of an mBC‐specific values assessment tool. The strengths of this tool include allowing for shifting values over time and enhanced rigor achieved through the guidance of a Community Advisory Board. The values assessment tool will be further tested to evaluate its impact on communication with clinicians and health outcomes for women with mBC.

## Author Contributions

L.A.C. conceived the study and study design. M.B. and L.A.C. contributed to data collection. R.M.F., V.C., and L.A.C. contributed to the data analysis. All authors contributed content expertise. V.C. and L.A.C. contributed to the first draft of the manuscript, which the authors edited. All authors read and approved the final manuscript.

## Conflicts of Interest

The authors declare no conflicts of interest.

## Data Availability

The data that support the findings of this study are available from the corresponding author upon reasonable request.

## Appendix A Interview Guide for Concept Elicitation

Section 1: Establishing a Context for a Discussion With Patients Who Have Been Diagnosed With Metastatic Breast Cancer (5 min)

1. To begin, please tell me a little about your cancer care team. How would you describe them?
  - How long have you been under their care?

Section 2: Factors That Influence Treatment Plan Decision‐Making (15 min)

2 What type of cancer treatment are you receiving or have you completed?

I want to ask you about your experience making treatment decisions for your breast cancer. Think back to when you were deciding on what treatment to pursue.

3 Tell me how the decision about your breast cancer treatment plan was made.
  - Who was involved in the decision about your treatment plan?
  - Who made the final decision?
  - How prepared did you feel to make this decision? (less interested in concrete information to make the decision and more curious if patients felt they understood what was important to them during the diagnosis and treatment discussion)
  - What, if anything, made it difficult when you were deciding about your treatment?
4 [KEY question] What was most important to you when you decided on your treatment plan? Were there other factors that you now would consider necessary when making the decision but didn't think about when you decided on your treatment plan?

Section 3: Communicating Experience Around Treatment Plan Decision‐Making (15 min)

5 How did your provider discuss your treatment options with you?
  - What were you thinking about or feeling during this conversation?
  - Was there any other information you wanted your clinician to share with you—if so, what would it have been?
  - What questions did you have for the care team when you were discussing treatment options?
  - What questions, in retrospect, do you wish you had asked?
6 [KEY Question] Did your clinician ask you about what was important/valuable to you?
  - How did your clinician ask you what was important/valuable to you?
  - How did you communicate that information to your clinician?
  - Did you feel they understood your values and or the important things to you?
  - Do you feel like telling your clinician what was most important to you changed the discussion about the treatment plan in any way?
  - How do you feel your treatment plan would change if you told the provider what was most important to you?
7 [KEY Question] How did your family member(s), caregiver(s), or other support influence your treatment decisions?
  - What did they (family/caregiver/support) say or do that made your decision easier?
8 What else impacted how you thought about and made decisions regarding your cancer treatment?
  - If not discussed, probe what impact faith/religion had on treatment decisions. Culture/ethnicity? Financial considerations?

Section 4: Closing (5 min)

9 Is there anything else you feel would be helpful for cancer care teams to know as they try to help patients like yourself make decisions about breast cancer treatment?

## Appendix B Follow‐Up Interview Guide

We would like to hear whether we have identified all the essential considerations. I will list them for you and then ask if we have missed anything or anything is not on target.

What is important to women with metastatic breast cancer when deciding on treatment? Everyone wants effective treatment, but what other considerations are important when making treatment decisions?

1. Quality of life during treatment
2. Living to care for children/grandchildren
3. Side effects of treatment
4. Desire to not appear sick
5. Stop or slow disease progression with an effective treatment plan that includes the patient.
6. Desire to help other patients by participating in clinical research
7. Lack of regret for decisions (fear of recurrence) (unsure) (Not endorsed by any follow‐up interviews)
8. Sexuality and impact on intimate relationships

Which of these stands out to you in your own experience with metastatic breast cancer?

- Probe: What about the statement stands out to you?
  ∘ How would you define it?
- Probe: How did this concept impact your decision‐making?

Thinking about your own experiences, is there anything missing from this list that was important to you when deciding your treatment options?

When you first heard the diagnosis of metastatic breast cancer, which of these concepts was most important to you?

Why?

Has that changed over time?

What is most important to you now?

Why do you think that has/has stayed the same?
